# Comparison of Typical Alpine Lake Surface Elevation Variations and Different Driving Forces by Remote Sensing Altimetry Method

**DOI:** 10.3390/ijerph192417090

**Published:** 2022-12-19

**Authors:** Yaming Pan, Weibing Du, Dandan Ma, Xiaoxuan Lyu, Chaoying Cheng

**Affiliations:** 1 School of Surveying and Land Information Engineering, Henan Polytechnic University, Jiaozuo 454003, China; 2 State Key Laboratory of Desert and Oasis Ecology, Xinjiang Institute of Ecology and Geography, Chinese Academy of Sciences, Urumqi 830011, China; 3 Xinjiang Key Laboratory of Remote Sensing and Geographic Information System Application, Urumqi 830011, China

**Keywords:** alpine lake, lake surface elevation, driving forces, CryoSat-2

## Abstract

Alpine lakes play a significant role in improving watershed ecology, adjusting water storage, and managing regional water resources. They are also a valuable freshwater reservoir, flood storage, and species gene pool in Central Asia. This article validated the accuracy of the CryoSat-2 footprints altimetry dataset for the Lake Bosten and Lake Issyk-Kul ranges. The time series for the surface elevations of the Central Asian alpine lakes Karakul and Chatyrkul were established, based on footprints altimetry data. The lake hydrological drivers were analyzed using remote sensing meteorological reanalysis data of the lake basins. The following main conclusions were reached. The CryoSat-2 footprints altimetry dataset has high confidence in lake surface elevation monitoring. Compared with Hydroweb monitoring results, the agreement between the monitoring results in the range between Lake Bosten and Lake Issyk-Kul are 0.96 and 0.84. The surface elevation of Lake Karakul shows an overall increasing trend with a variation rate of +7.7 cm/yr from 2010 to 2020, which has a positive correlation with the temperature in the basin. This indicates that the increased temperature, which results in the increased snow and ice meltwater in the basin, is the main driving force of the increased lake evolution. The lake surface elevation of Lake Chatyrkul shows an overall decreasing trend, with a variation rate of −9.9 cm/yr from 2010 to 2020, which has a negative correlation with the temperature in the basin. This suggests that Lake Chatyrkul is poorly recharged by snow and ice meltwater. The main driving force of its evolution is the increased evaporative output of the lake due to the increase in temperature. These conclusions prove that temperature and alpine glacial variability within the lake basin play an important role in lake surface elevation variations in alpine regions of Central Asia.

## 1. Introduction

As an important part of the global hydrological and biogeochemical water cycle, lakes play an important role in runoff regulation, water recharge, and the ecological balance of watersheds [1,2,3]. Their formation and disappearance, expansion and contraction, and evolution of the surrounding ecosystem are the results of complex interactions of global, regional, and local tectonic and climatic events [4]. Especially in the arid and semi-arid environments of Central Asia, lakes are more vulnerable to changes in the surrounding climate and environment [5]. Changes in lake levels and storage volumes have significant aquatic and ecological impacts on a local and regional scale [6]. Therefore, long-term, accurate monitoring of Central Asian alpine lakes and their surroundings is urgently needed.

Traditional lake monitoring methods rely on “direct” measurements or monitoring through hydrological stations [7]. The method has high monitoring accuracy in a small monitoring range. However, the complex topography and variable weather conditions in alpine areas limit the long-term measurement and observation of some lakes, especially in alpine areas where meteorological and hydrological stations are difficult to establish and maintain. Thus, monitoring lakes in alpine areas using traditional methods becomes extremely difficult. Based on this research background, satellite remote sensing technology, which can easily obtain long-term and high-precision monitoring data, has become an important way to observe lakes in alpine regions [8,9,10,11,12,13,14]. Currently, many organizations have deployed short revisit cycle altimetry satellites to dynamically monitor globally important water bodies, such as Hydroweb, established in 2011 by the French Space Geophysics and Oceanography Research Laboratory (LGOR) [15]; the European Space Agency’s (ESA) Rivers and Lakes project in 2012; and the Database of Hydrological Time Series of Inland Waters (DAHITI), constructed by the German Geological Institute [11]. Remote sensing meteorological products also play an important role in predicting future trends in the evolution of lakes [16,17,18,19,20].

Current studies on lakes in the alpine region of Central Asia are mainly focused on the Qinghai–Tibet Plateau region. Wang has explored the average ice phenology and evaporation of 75 large glacial lakes on the Qinghai–Tibet Plateau by combining meteorological and satellite data and has elaborated the spatial distribution and influencing factors of evaporation in the region, providing important support in the study of regional climate change impacts and water resources management [21]. To predict future water level change trends, scholars have also used CryoSat-2 altimetry satellite data to monitor the water level change cycles of more than 200 high mountain lakes in the Tibetan Plateau region. According to their calculations, the water level growth rate in the region can be monitored with an accuracy of 2 cm level [22]. Previous studies have combined CryoSat-2 and ICESat-2 satellite altimetry data with Landsat and Gaofen series optical images to obtain water level and water storage variations of lakes on the Qinghai–Tibet Plateau, Gahai Lake, Dianchi, and other places. Results indicate that the water level and water storage of lakes in the region continue to grow, and the monitoring accuracy can reach the centimeter level [19,20]. Those work also reflects that multi-source altimetry satellites including CryoSat-2 can be used for monitoring anomalous hydrological conditions of high mountain lakes, and analyzing spatial and temporal differences in lake elevation variations on the Tibetan Plateau. It provides important data support for lake disaster warning and watershed ecosystem research [21].

In the alpine region of Central Asia, there are still some typical alpine lakes that need additional monitoring; their data need to be updated continuously, and their drivers need to be analyzed. Especially in the Pamir Plateau region, there are fewer studies about the alpine lakes in this region, and there is a lack of research works such as time series reconstruction of lake surface elevation and analysis of lake drivers in recent years.

In this paper, we aim to reconstruct the water levels of typical alpine lakes in Central Asia based on altimetric satellite data, to determine the dynamic trends of the lakes, and to discuss the main drivers of lake changes based on remotely sensed meteorological products. First, we verified the accuracy of CryoSat-2 altimetry satellite data; then, based on this, we established the surface elevation change curve of the lake from 2010 to 2020. Then, the main driving forces of the lake changes were analyzed based on ERA5 remote sensing meteorological products. In addition, a new method to remove anomalous elevation points of alpine lakes for satellite altimetry data is constructed in this paper for obtaining surface elevations of alpine lakes in Central Asia.

## 2. Materials and Methods

### 2.1. Study Area

Lake Karakul’s center is located at 39° N, 73.41° E. It is located in Tajikistan’s Pamir Plateau’s eastern mountain range at a height of roughly 3890 m. It is 19 km long from east to west and 27 km wide from north to south. Lake Karakul covers an area of about 400 km^2^ with an average water depth of about 15 m. The surface of the lake can be covered with ice from November to May each year due to the low winter temperatures, and the ice can be up to 1 m thick. The average annual precipitation is about 82 mm [23,24,25]. The lake is a closed basin lake, and the recharge of the lake mainly comes from glacial meltwater and snow meltwater, which is a typical ice and snow ablation recharge type lake.

Lake Chatyrkul’s center is located at approximately 40.60° N, 75.33° E, between the ridges of At-Bashi and Torugart-Too, Kyrgyzstan, with an elevation of about 3500 m. It is approximately 26 km long from east to west, and 16 km wide from north to south. The lake covers an area of 175 km^2^, with a depth of about 13 m. It is the third largest lake in Kyrgyzstan, and it is frozen for most of the year. The lake basin is a closed basin, and it is recharged mainly by about 14 surrounding rivers.

Lake Bosten is located at 42.08° N, and 87.03° E. It is located in Bohu County, Xinjiang Uygur Autonomous Region, China, with an altitude of about 1048 m. It is about 55 km long from east to west and 25 km wide from north to south. The water area of the lake is about 1228 km^2^. The water storage is about 8.8 km^3^, and the depth of the lake is 9~17 m. The surface of the lake is covered by ice from January to March every year, and the annual precipitation is about 62 mm. The lake is the largest inland freshwater lake in China, and it belongs to the mountain trap lake. Its main recharge river is the Kaidu River, and it is the main source of the Peacock River. Its source of recharge mainly comes from the glacier ice meltwater in the Tianshan region [26,27].

Lake Issyk-Kul’s center is located at 42.43° N, 77.33° E, in the territory of Kyrgyzstan, north of the Tian Shan Mountains. The altitude is about 1609 m. The length from east to west is about 178 km, and the width from north to south is about 60 km. Lake Issyk-Kul has an area of 6236 km^2^, a storage capacity of 1378 km^3^, and a depth of about 300–700 m. It is a year-round unfrozen lake with annual precipitation of about 200–300 mm [28]. Lake Issyk-Kul is the largest alpine saltwater lake in Central Asia. It is recharged mainly by surface rivers and groundwater originating from the glaciers and snowfields of the Tian Shan Mountains [5,29,30].

**Figure 1:**
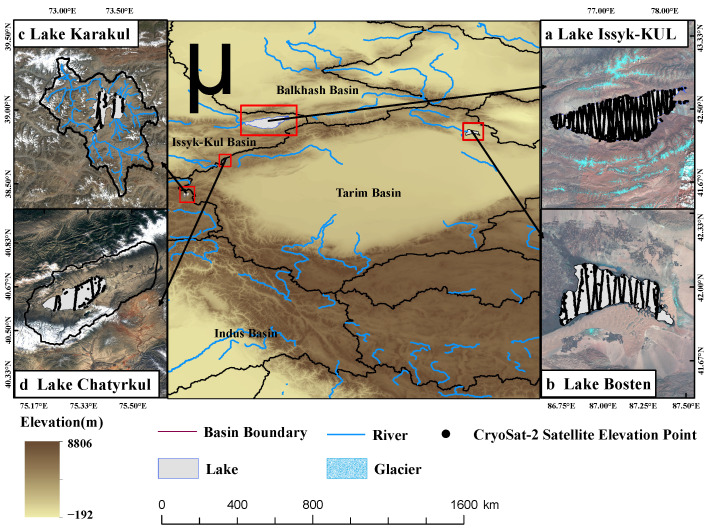
The geographical location of the lakes in the study area.

As shown in Figure 1, both Lake Karakul and Lake Chatyrkul are located in the alpine region of Central Asia, but both their elevations and areas covered by glaciers are different. The differences between the two may reflect the influence of elevation and glaciers on alpine lakes. However, since most of the meteorological stations established around these two lakes stopped working before 2000, the recent work on water level and climate observation is lacking, and there are no sufficient data to analyze the overall trend of the lakes.

The continuous monitoring of water levels in Issyk-Kul and Bosten lakes has been relatively well established, and its measuring accuracy is relatively accurate. The following study compares the time series of lake water levels, obtained from CryoSat-2 satellite altimetry data, with the benchmark of lake water levels, collected from the Hydroweb website, to verify the accuracy of the altimetry satellite data in monitoring the trend of lake water levels. 

### 2.2. Data

#### 2.2.1. Lake Surface Elevation Data

In this study, two main types of lake elevation data were used to monitor the surface elevation variations of Central Asian alpine lakes.

(1) CryoSat-2 altimetry satellite was launched in 2010. It carries a synthetic aperture interferometric radar altimeter (SIRAL), which runs in Ku-band and has three operation modes: low resolution mode (LRM), synthetic aperture mode (SAR) and synthetic aperture interferometric mode (SARIn) [31]. The SARIn mode is mainly used in mountainous areas and at the edge of ice caps, which can effectively monitor water level variations and has a high spatial coverage in Central Asia. In this mode, the altimeter performs synthetic aperture processing along the orbit with an extended range sampling window. It uses a second antenna as an interferometer to determine the orbital angle of the earliest radar return by interferometry, which means that the CryoSat-2 satellite can provide more reliable water level data for lakes surrounded by complex terrain [32,33].

This study used the CryoSat-2 Level 2 (L2) Baseline-D product, which can provide better results for lake surface elevation monitoring than the previously released Baseline-C product. The CryoSat Ice Baseline-D processor generates Class 1B and Class 2 products from Class 0 LRM, SAR, and SARIn products. In all three modes, the Level 1B data include echoes from every point in the satellite’s ground orbit, and the data all consist of multicolor echoes at a rate of approximately 20 Hz; the Level 2 product contains surface elevation measurements, which is fully corrected for instrumental effects, propagation delays, measurement geometry, and other geophysical effects such as atmospheric and tidal effects [34]. L2 products are generally considered to be the most user-friendly products. The raw data for the L2 product are global altimetric points with a monthly cycle. In this study, related CryoSat-2 satellite footprint data from July 2010 to June 2021 are extracted by Matlab.

(2) In this study, time series data of water levels in the world’s rivers and lakes from the Hydroweb website (https://hydroweb.theia−land.fr/, accessed on 6 March 2022). The Hydroweb database of water level data are a combination of multi-source altimetry satellite data from different service years, including TOPEX/Poseidon, ERS-1 and ERS-2, Envisat, Jason-1, and GFO satellites. This constantly updated database provides water level and area monitoring results for major lakes, reservoirs, and rivers worldwide [15]. This dataset is mainly used as validation data in this study to demonstrate the accuracy of the CryoSat-2 satellite altimetry dataset for lake surface elevation monitoring.

#### 2.2.2. Meteorological Data

ERA5 is the fifth generation of global climate reanalysis data, and it is published by the European Centre for Medium-Range Weather Forecasts (ECMWF). The ERA5-LAND (ECMWF) climate reanalysis data uses the laws of physics to combine model data with global climate observations into a complete and consistent dataset. It provides data about the evolution of land variables over decades at a higher resolution. The dataset is recorded from 1981 with a spatial resolution of 11,132 m. It has an hourly temporal resolution, which provides an accurate description of past climate. The dataset contains multiple variables describing water and energy cycles over land [35,36,37]. The variables “temperature_2m” and “total_precipitation” in the dataset are used to monitor temperature and precipitation trends in the lake basin.

### 2.3. Methodology

#### 2.3.1. Surface Elevation Extraction of Lakes

This study mainly used the CryoSat-2 satellite altimetry dataset, whose main observation principles are as follows:

(1) E=alt−range+c

where E is the distance from the lake surface to the WGS-84 reference ellipsoid, i.e., the lake surface elevation; alt is the distance from the satellite to the WGS-84 reference ellipsoid; the range is the distance from the satellite to the lake surface; and c is the correction value, including geophysical corrections (solid Earth tides, ocean loading tides, and geocentric polar tides) and atmospheric corrections (ionosphere, wet troposphere, and dry troposphere).

In this study, L2-level products were used for the calculation of lake surface elevation. In addition, the lake surface elevation time series reconstruction was assisted by extracting the longitude, latitude, and acquisition time from the satellite footprints in the dataset. CryoSat-2 satellites are based on radar altimeter measurements of surface elevation, and data quality can be affected by various factors (cloud cover, rainfall, lake reflections, and satellite footprint size), resulting in anomalous footprints. To solve this problem, two statistical methods are widely used, namely the mean value method and the “Pauta Criterion” method [22,38,39]. The mean value method calculates the mean value of the lake elevation footprints directly. It takes the result as the final elevation of the lake, but the final result is greatly influenced by anomalous values. The “Pauta Criterion” method only accepts the points, whose remaining errors are within three standard deviations, as valid footprints; the rest of the elevation points are rejected as outliers. This method is more accurate in estimating elevation values of the lake surface. However, when the number of anomalous footprints in the set of all footprints is large, this method does not work well to reject anomalous footprints. In order to improve the “Pauta Criterion” method, we propose a new method based on it. The proposed method combines the idea of lake boundary and threshold extraction; it filters and extracts the footprint data of Central Asian alpine lakes, and then the average value of footprint elevation is used as the surface elevation of the lake.

In this study, the lake surface elevation was extracted based on the two assumptions that the lake surface is approximately horizontal and the lake surface elevation points are normally distributed, and the anomalous footprints are removed.

In order to reduce the influence of shore features on satellite footprints, buffer zone analysis was performed on the lake boundary vector. The lake boundary line was pushed inward by 200 m to obtain the elevation point extraction range “Range”; the CryoSat-2 footprints dataset was extracted using “Range” as the range reference; and the extracted dataset was aggregated by month according to the data collection time to obtain the original lake elevation point set EP_1_ (p_1_,p_2_,…, p_i_), EP_2_ (p_1_,p_2_,…, p_i_), EP_i_ (p_1_,p_2_,…, p_i_).

First, for each original footprint set EPi, the anomalous footprints of the footprint set are eliminated using the idea of threshold extraction, the principle is as follows:

(2) $$p_{i}\in \{\begin{array}{c} {\mathrm{List}}_{i}\;,\;\;\;\left|S_{i}-p_{i}\right|<t\\ \mathrm{Outliers},\;\left|S_{i}-p_{i}\right|\geq t \end{array}$$

where p_i_ is the original set of footprints in EP_i_, S_i_ is the median value of footprints in EP_i_, t is the set elevation error threshold, Listi is the set of footprints in EP_i_ after eliminating obvious anomalies, and outliers is the set of anomalous footprints to be eliminated.

In order to further improve the elevation measurement accuracy and reduce the effect of coarse differences, this study rejects each footprint set List_i_(p_1_,p_2_,..., p_i_) based on the Lajda criterion with the following equation:

(3) $$p_{i}\in \{\begin{array}{c} {\mathrm{EEP}}_{i},\;\;\;\;\left|p_{i}-\mu_{i}\right|<3\sigma_{i}\\ \mathrm{Outliers},\left|p_{i}-\mu_{i}\right|\geq 3\sigma_{i} \end{array}$$

where p_i_ is the footprint in the footprint set List_i_, EEP_i_ is the final set of valid footprints corresponding to the set List_i_, μi is the mean value of elevation corresponding to the footprints in the set List_i_, σ_i_ is the standard deviation corresponding to the footprints in the set List_i_, and outliers are the anomalous footprints for rejection. The average elevation value of each effective footprint set EEP_i_ is finally calculated as the lake surface elevation during the observation period.

Through this method, CryoSat-2 satellite altimetry data were processed; the number of points within each EP and EEP set was counted at the same time; the sets whose number of footprints were both less than a certain threshold were eliminated; the lake surface elevation was calculated for each time, and a time series was finally established.

**Figure 2:**
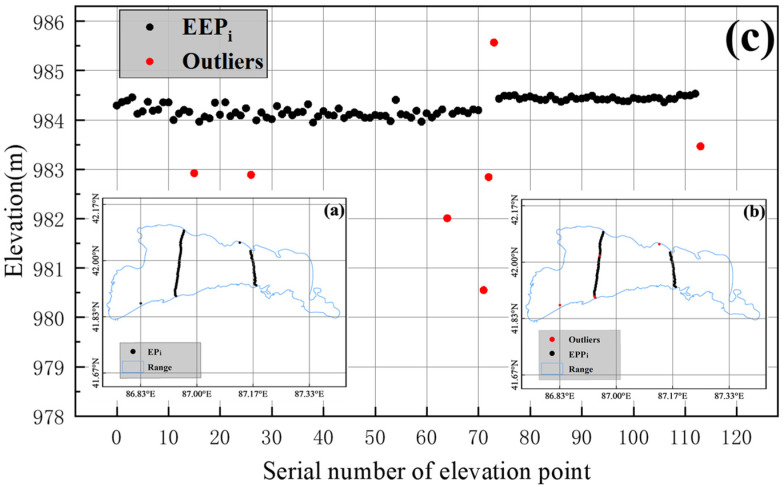
June 2015 data as an example. (**a**) Horizontal distribution of original footprints EP_i_ in Bosten Lake. (**b**) Horizontal distribution of effective footprints EPP_i_ and anomalous footprints outliers. (**c**) Vertical distribution of valid footprints EPP_i_ and anomalous footprints outliers.

Figure 2 demonstrates the effect of this method to reject anomalous values. Figure 2a shows the spatial distribution status of the original CryoSat-2 satellite footprints within Lake Bosten in June 2015. Figure 2b shows the spatial distribution of the anomalous footprints within the lake during the same time. From Figure 2b, it is observed that the anomalous footprints are generally distributed on the shore of the lake, which indicates that the shore features have a certain influence on accurate lake measurement. Figure 2c shows the distribution of footprints in the vertical direction within the lake. As shown in Figure 2c, after eliminating the anomalous footprints (red dots in the figure), the distribution of effective footprints of the two trajectories is approximately a straight line in the vertical direction, which agrees with the natural law that the surface of the lake is approximately a plane. This finding indicates that the method can effectively eliminate anomalous footprints in the satellite footprints data.

#### 2.3.2. Basin Climate Variable Extraction

This study used the GEE (Google Earth Engine) platform for data acquisition and processing and analyzes the climate factors (temperature and precipitation) within the lake basin based on ERA5 remote sensing climate reanalysis data. The acquired remote sensing climate reanalysis raster data was extracted by mask basing on the lake basin boundary to obtain basin temperature and precipitation raster data. Each pixel point of the raster data represents the total precipitation or average temperature within that point. Each pixel value in the basin raster map was statistically analyzed to obtain cumulative precipitation totals and average temperatures at the basin scale every month.

**Figure 3:**
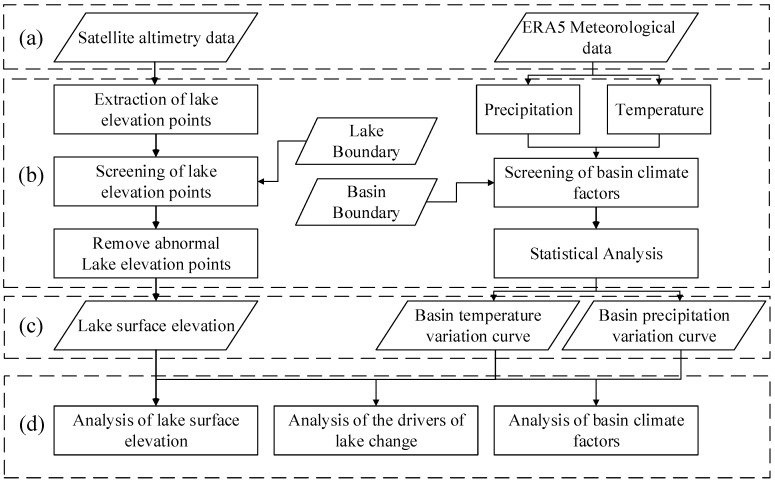
Technical flow chart. (**a**) Data source. (**b**) Data processing process. (**c**) Lake surface elevation and basin temperature precipitation profiles. (**d**) Trend and driver analysis of lake variability.

Figure 3 shows the main workflow of this study, which is divided into four main parts, respectively (a) Data source, (b) Data processing process, (c) Lake surface elevation and basin temperature precipitation profiles, and (d) Trend and driver analysis of lake variability.

## 3. Results

### 3.1. Verification of Elevation Extraction Accuracy

The reference datum of the two lake elevation data used in this study is different, Hydroweb reference surface uses GGMO2C elevation datum and CryoSat-2 uses EGM96 elevation datum, so there is some difference between Hydroweb data and CryoSat-2 elevation values. This study eliminates the effect of this difference by elevation conversion and performs linear regression analysis and consistency analysis on the two elevation data.

**Figure 4:**
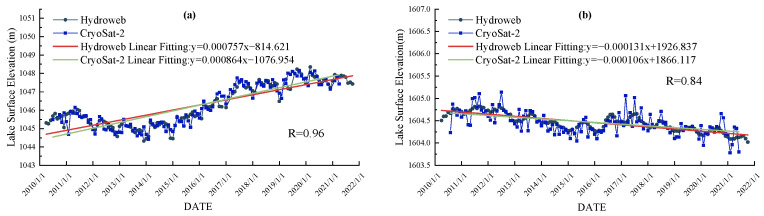
(**a**) Time series of the surface elevation of Lake Bosten based on CryoSat-2 satellite and Hydroweb data. (**b**) Time series of the surface elevation of Lake Issyk-Kul based on CryoSat-2 satellite and Hydroweb data.

As shown in Figure 4, the time series trends of lake surface elevation of Lake Bosten and Lake Issyk-Kul from 2010 to 2020 obtained by CryoSat-2 altimetry satellite monitoring are the same as the monitoring data in Hydroweb, and the agreement between the two data on the two lakes is 0.96 and 0.84, respectively, which confirms the credibility of CryoSat-2 altimetry data. This indicates that the data can be used for lake surface elevation monitoring and the measurement accuracy can meet the demand.

### 3.2. Surface Elevation Extraction of Central Asian High Mountain Lakes

**Figure 5:**
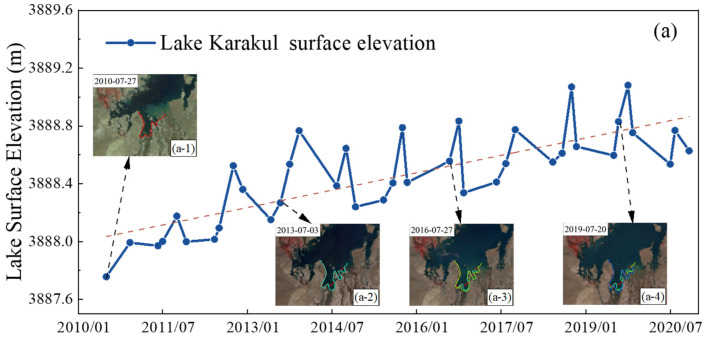

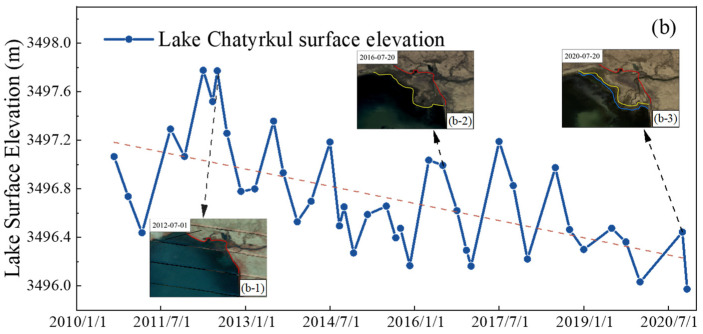
(**a**) The surface elevation variation curve of Lake Karakul. (**b**) Surface elevation variation curve of Lake Chatyrkul.

In this study, the time-series reconstruction of lake surface elevation is carried out for two High Mountain lakes (Lake Karakul and Lake Chatyrkul). The CryoSat-2 satellite footprints data of the two lakes are processed according to the lake surface elevation extraction algorithm proposed in 3.2.1. Figure 5a,b show the satellite footprints dataset of these two lakes’ monthly variations in lake surface elevation.

From Figure 5a, it can be seen that the surface elevation of Lake Karakul shows an overall rising trend from 2010 to 2020, fluctuating between 3887.75 m and 3889.08 m, showing a small increasing trend of 7.7 cm/yr. The lake boundary is slowly expanding outward. Figure 5(a-1,a-2,a-3,a-4) show the southwestern boundary of the lake on 27 July 2010, 3 July 2013, 27 July 2016, and 20 July 2019; and they are respectively shown with red, cyan, green, and blue lines to present the expansion of the lake. Combining the variations in the lake area and surface elevation, it is analyzed that the water volume of Lake Karaikul increased by about 3.7 × 10^6^ m^3^ from 2010 to 2020. The surface elevation of this lake had three anomalous decline years (2014, 2017, and 2020) and the lake level declined by 0.12 m, 0.06 m, and 0.32 m, respectively, with the most dramatic decline in 2020. The seasonal variation in the surface elevation of Lake Karakul is small, and its variation does not exceed 1 m during the year. Additionally, the overall trend of the surface elevation of Lake Karakul is relatively stable. Overall, Lake Karakul is in a fluctuating rising trend.

Compared with Lake Karakul, which is about 414.25 km^2^, Lake Chatyrkul is smaller, which is about 257.12 km^2^. As a result, the CryoSat-2 satellite landed less satellite footprint data in this area, and several monthly footprints data are missing. In this study, when constructing the surface elevation time series of Lake Chatyrkul, it is found that there are obvious abnormal deviations in elevation data between several months and their neighbors. By analyzing these original footprint data, it is found that in the months with abnormal elevation, there is a smaller number of lake footprints; moreover, a lot of these footprints are abnormal. This indicates that the accuracy of elevation measurement is poor in these particular months, so the water level data of these months should be directly removed from the lake surface elevation time series.

Figure 5b shows that Lake Chatyrkul has an overall decreasing trend in lake surface elevation from 2010–2020, from 3497.07 m to 3495.97 m, with a variation rate of −9.9 cm/yr. The boundary of Lake Chatyrkul slowly recedes inward. Figure 5(b-1,b-2,b-3) show the northeastern boundary of the lake on 1 July 2011, 20 July 2016, and 20 July 2020, respectively; they are shown as red, yellow, and blue lines, which agree with the fact that the lake is retreating. Combining the variations in the lake area and surface elevation, it is concluded that the volume of Lake Chatyrkul decreases by about 1.7 × 10^6^ m^3^ from 2010 to 2020. Overall, Lake Chatyrkul is in a fluctuant downward trend as a whole.

## 4. Discussion

The lake itself is a dynamic system. In other words, under the influence of the surrounding landscape and anthropogenic pressures, it can show its significant responses to local climatic and non-climatic variables [40]. Surface water inflow and outflow, evaporation from the lake surface, and water use for human production and living are all major factors in the variations of the lake [41,42]. In alpine areas, lakes are less affected by human production and life; variations in lake water are more likely to reflect trends in surrounding climate change [43]. Thus, alpine lakes can serve as an indicator of the local climate within the watershed.

Terms of temperature are affected by altitude in alpine regions. The higher the altitude is, the lower the temperature is. The annual temperature extremes can better reflect the trend of temperature variation in the alpine lake basin. That is, the month with the highest temperature generally belongs to the yearly glacial ablation period, and the month with the lowest temperature generally belongs to the yearly glacial accumulation period [44]. In other words, under certain circumstances, the annual temperature extremes in the basin could reflect the glacial ablation situation in the basin. In terms of precipitation, it is an important water recharge in the evolution of the lakes, and it plays an important role in the study of the drivers of lake variation. In addition, the annual cumulative precipitation reflects the total precipitation of the region in a year. 

In closed watersheds with low human activities, lake surface elevation variations are mainly related to water recharge. At present, the effect of precipitation on inland lake levels is mainly considered to be positive, in contrast, the effect of temperature on inland lake levels is mainly considered to be negative. The reason is that the increased precipitation directly increases the water recharge of the lake, which leads to an increase in lake input; the increased temperature enhances the evapotranspiration of the lake, which leads to an increased lake output [45,46,47,48]. However, in alpine areas of Central Asia, the temperature also contributes to the glaciers and snow meltwater in the basin, which takes into account the recharge of lake water volume [40,44,49]. For example, it has been shown that there is a positive effect of temperature on alpine lakes in the Qinghai–Tibet Plateau region [50,51,52]. Therefore, for high mountain lakes, especially small lakes with a large number of glaciers in the lake basin, the role of temperature on the surface elevation of the lake needs to be further analyzed.

Correlation analysis provides a better response to the influence of different variables on the surface elevation of the lakes [21,53]. In this study, temperature and precipitation in the basins of Lakes Karakul and Chatyrkul for 2010–2020 were counted. By analyzing the correlations between these variables and lake surface elevation, the main drivers of the two lakes were explored and the intrinsic reasons affecting the different drivers of the lakes were investigated.

### 4.1. Analysis of the Main Drivers of Lake Karakul

**Figure 6:**
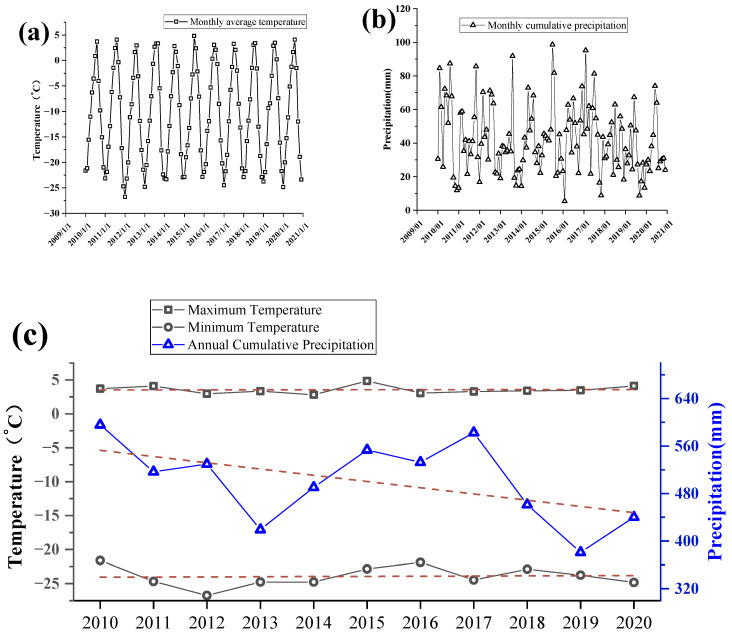
(**a**) Monthly average temperature variation curve in the Karakul Lake basin from 2010 to 2020. (**b**) Monthly cumulative precipitation curves in the Karakul Lake basin from 2010 to 2020. (**c**) Annual temperature extremes and annual cumulative precipitation curves in the Karakul Lake basin from 2010 to 2020.

As shown in Figure 6, in terms of precipitation, the overall trend of precipitation within the Karakul Lake basin is increasingly arid. The annual cumulative precipitation decreases from 595.69 mm in 2010 to 440.28 mm in 2020. For seasonal differences in precipitation, the annual precipitation in the Karakul Lake basin is mainly concentrated from July-October, but even in the months when precipitation is concentrated, the total precipitation in the basin is only about 80 mm. This indicates that the lake basin is in a semi-arid state. In terms of temperature, the lake is at a high altitude. The annual maximum temperature of the Karakul Lake basin is only about 5 °C during 2010–2020, and the minimum temperature reaches −25 °C. The annual extremes of the basin temperature are generally in a slow upward trend.

**Figure 7:**
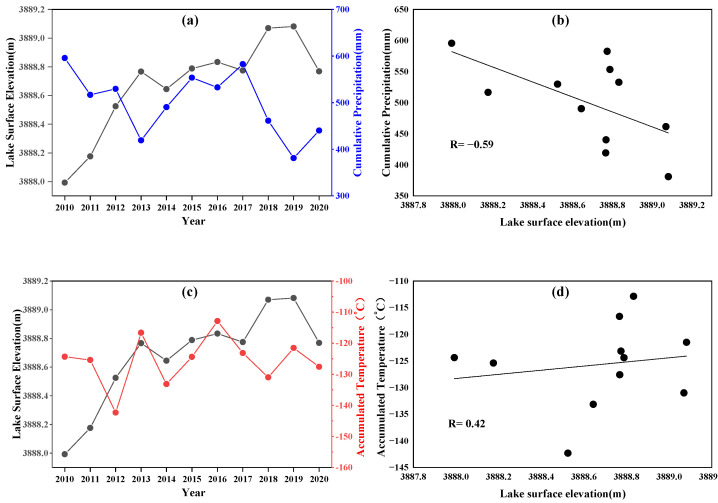
(**a**) Annual cumulative precipitation and lake surface elevation curves of Lake Karakul. (**b**) Correlation between cumulative precipitation and lake surface elevation. (**c**) Annual cumulative temperature and lake surface elevation curves of Lake Karakul. (**d**) Correlation between cumulative temperature and lake surface elevation.

Figure 7 shows the relationship between the lake surface elevation of Lake Karakul and the precipitation, and the relationship between the lake surface elevation of Lake Karakul and the temperature in the basin from 2010 to 2020. The figure reflects that the overall surface elevation of Lake Karakul has had a fluctuating upward trend over the years, which shows a relatively strong negative correlation with the precipitation in the area (R = −0.59). It indicates that the surface elevation of Lake Karakul did not decrease with the decrease in precipitation in the basin, and precipitation was not the main driving factor for the fluctuating increase in the surface elevation of this lake during 2010–2020.

The annual accumulation temperature is calculated as the sum of temperatures for 12 months of the year, representing the heat accumulation in the region. To some extent, it reflects the glacial melt and lake evapotranspiration variations in the region. As shown in Figure 7d, there is a positive correlation between the surface elevation of Lake Karakul and the annual accumulated temperature in the basin, but the correlation is poor. The main reason is that the effect of temperature on the surface elevation of the lake is twofold. On the one hand, the increased temperature increases the evapotranspiration of the lake, which leads to an increase in lake output; on the other hand, the increased temperature promotes the melting of glaciers and snow in the basin, which leads to an increase in surface runoff and an increase in lake input.

Glacier mass loss exists in the eastern Pamir Plateau, and glaciers are melting in some areas [54,55]. In this study, we looked for glaciers within the Lake Karakul basin and found that glaciers are continuously melting within the basin [56]. Based on the positive correlation between temperature and lake surface elevation, and combined with the mentioned fact that precipitation is not the main driver of the steady growth of Lake Karakul, it is suggested that the recharge of ice and snow meltwater from increasing temperature has the main driving effects on the rise of lake surface elevation.

In summary, the surface elevation of Lake Karakul shows a fluctuating rising trend, and the precipitation in the basin shows a fluctuating decreasing trend. The main driving factor of its lake evolution is the recharge of increased glacial snow meltwater due to the increased temperature.

### 4.2. Analysis of the Main Drivers of Lake Chatyrkul

**Figure 8:**
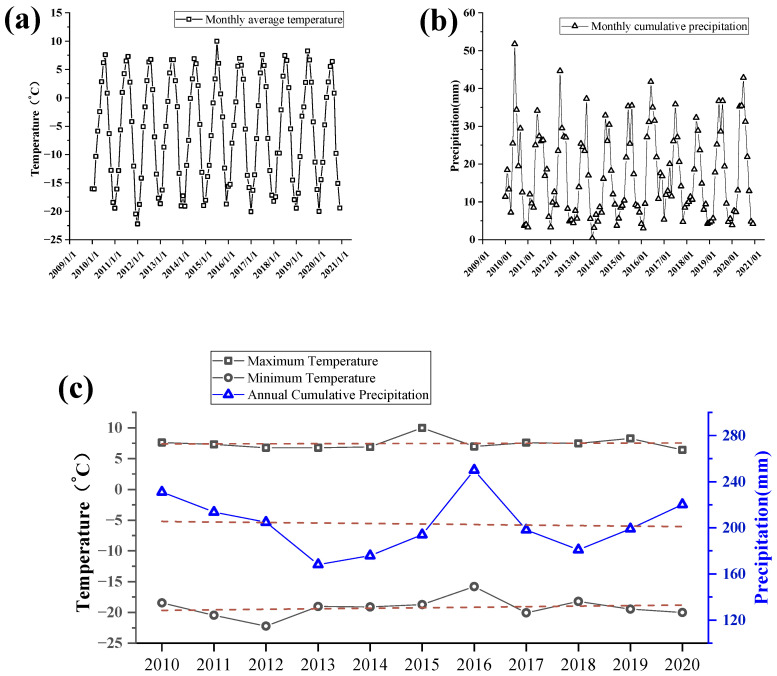
(**a**) Curves of monthly average temperature variations in the Lake Chatyrkul basin from 2010 to 2020. (**b**) Curves of monthly cumulative precipitation in Lake Chatyrkul basin in 2010–2020. (**c**) Curves of annual temperature extremes and annual cumulative precipitation in the Lake Chatyrkul basin in 2010–2020.

Compared with the Lake Karakul basin, the Lake Chatyrkul basin is smaller. As shown in Figure 8, in terms of precipitation, the cumulative precipitation in its basin is low, and the annual cumulative precipitation fluctuates between 168.17 mm and 250.01 mm, with no obvious trend of increase or decrease. For seasonal differences, the annual precipitation in the Lake Chatyrkul basin is mainly concentrated from June to September, with precipitation of about 43 mm. The overall precipitation in this lake basin is low and basically in a dry state. In terms of temperature, the annual maximum temperature in the Lake Chatyrkul basin from 2010 to 2020 is around 7.5 °C, and the minimum temperature is around −20 °C, with an overall slow increase.

**Figure 9:**
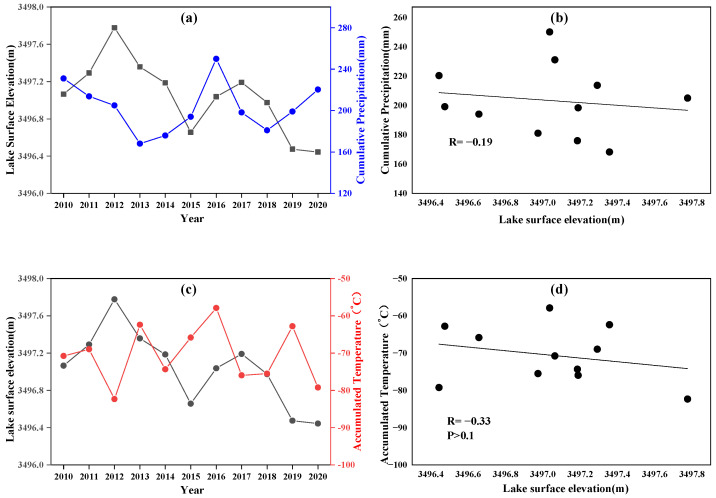
(**a**) Annual cumulative precipitation and lake surface elevation curves for Lake Chatyrkul. (**b**) Correlation between cumulative precipitation and lake surface elevation. (**c**) Annual cumulative temperature and lake surface elevation curves of Lake Chatyrkul. (**d**) Correlation between cumulative temperature and lake surface elevation.

Figure 9 shows the surface elevation of Lake Chatyrkul from 2010 to 2020 and its relationship with precipitation and temperature. In 2010–2020, the surface elevation of the lake shows a fluctuating decreasing trend; the annual accumulated precipitation in the basin fluctuates significantly with a slight overall decrease; the correlation between the two sets of data is poor (R = −0.19). Combined with the positive effect of precipitation in the basin on the water recharge of the lake, this result indicates that precipitation is not the main driving factor for the evolution of Lake Chatyrkul. In terms of temperature, the temperature of Chatyrkul Lake basin does not have a clear upward or downward trend and is in a state of fluctuation, the surface elevation of Lake Chatyrkul shows a certain negative correlation (R = −0.33) with the annual accumulated temperature in the basin.

For Central Asian alpine lakes, glaciers in the basin play a crucial role in the evolutionary process of the lake. The current study of lake variations in the Hindu Kush–Karakorum–Himalayan range in Pakistan found that there was a positive correlation between the rate of lake expansion and altitude, with glacial melting playing a dominant role in the lake variation process [57,58]. Zhang et al. studied the water level variation pattern of lakes on the Qinghai–Tibet Plateau and found that there were some lakes with decreasing water levels. By analyzing climatic factors, it was found that in lakes with small basins, if there was not enough replenished water from other sources (glaciers or snow melt in the basin), usually the precipitation in the basin was not enough to compensate the water loss caused by evaporation from the lake, and the surface elevation of the lake would in a decreasing trend [58,59]. According to the glacier cover in the study area, the number and area of glaciers in the watershed of Lake Chatyrkul are extremely small. Thus, the aspect of temperature influence on Lake Chatyrkul, which can lead to glacial snow melt to recharge the surface runoff, is extremely limited; and the main aspect of temperature influence on Lake Chatyrkul is the evapotranspiration of the lake. This conclusion agrees with the negative correlation between temperature and lake surface elevation.

In summary, the effect of temperature on Lake Chatyrkul is different from that of Lake Karakul. The main effect is the lake surface evapotranspiration, which leads to the change in lake output, and the precipitation has less influence on the lake surface elevation of Lake Chatyrkul. In short, the main driver of the evolution of Lake Chatyrkul is the effect of temperature on lake evapotranspiration. If the temperature increases, the surface elevation of Chatyrkul Lake will decrease further.

### 4.3. Analysis of Differences in Lake Variation Patterns

Lake Karakul and Lake Chatyrkul are both alpine lakes, but they show different development trends. The surface elevation of Lake Karakul shows an overall increasing trend and Lake Chatyrkul shows a fluctuating decreasing trend. Comparing the differences between the two lakes, it is found that the surface elevation of Lake Karakul is about 400 m higher than that of Lake Chatyrkul, and the average difference in temperature is about −2.5 °C. Due to the difference in the area of the two lake basins, the spatial scale of the ERA5 remote sensing meteorological reanalysis data is used as the unit area to compare the average precipitation differences in per unit area in these two basins. The precipitation per unit area in the basin of Lake Karakul is about 9 mm lower than that in the basin of Lake Chatyrkul. The overall climatic differences show that Lake Karakul is more likely to be dry and cold, and Lake Chatyrkul is more likely to be hot and humid. According to the analysis in 4.1 and 4.2, the main driving factor of Lake KaraKul, which has a larger glacier area in its basin, is the increased ice meltwater recharge due to increased temperature. In contrast, the main driving factor of Lake Chatyrkul, which has a smaller glacier area in its basin, is the increased evapotranspiration on the surface of the lake due to the increased temperature. This result indicates that the glaciers around the alpine lakes in Central Asia play a significant role in the evolution of the lake.

In summary, for the Central Asian alpine lakes, precipitation is generally low in the basin. The influence of precipitation on lake variability is small, while temperature plays an important role in lake variability. In addition, based on the area covered by glaciers around the lakes, the effect of temperature on the lakes is different. For lakes surrounded by more glaciers in the basin, the effect of temperature on glacier ablation is greater than the effect of temperature on lake evapotranspiration. In other words, temperature generally shows a positive effect (Lake Karakul). In contrast, for lakes with fewer glaciers or no glaciers in the basin, the effect of temperature on the lake is mainly about evapotranspiration, which generally shows a negative effect on the surface elevation of the lake (Lake Chatyrkul).

## 5. Conclusions

The surface elevation of a lake is an important physical quantity to describe the hydrological condition of the lake. Based on CryoSat-2 point cloud altimetry data, this study proposes a method to eliminate anomalous footprints in the point cloud collection process, and extracts the lake surface elevation by combining the idea of lake boundary and threshold extraction. Based on the proposed method, the surface elevation change curves of typical alpine lakes in Central Asia, Lake Karakul, and Lake Chatyrkul were reconstructed from 2010 to 2020. Meanwhile, the main driving forces of the changes in the above lakes were analyzed in this study based on ERA5 remote sensing meteorological reanalysis data.

The results of this study indicate that the lake surface elevation of Lake Karakul variations at a rate of +7.7 cm/yr from 2010 to 2020, showing a fluctuating upward trend, and the meteorological change in the basin is trending toward “warm and dry”, the main drivers of lake variation are increased temperature and increased surface runoff recharge from increased glacial snow meltwater; the lake surface elevation of Lake Chatyrkul variations at a rate of −9.9 cm/yr from 2010 to 2020, showing a fluctuating downward trend, with no significant trend in meteorology in the basin, the main driver of the lake variation is an increase in temperature and an increase in lake output due to enhanced surface evapotranspiration from the lake itself.

The results of this study can help to complement studies on dynamic monitoring and trend prediction of typical alpine lakes in Central Asia. In Central Asia, especially in the Pamir Plateau region, the influence of precipitation on lakes is weak, and temperature is an important factor influencing the variability of lakes. It is worth noting that watershed temperature has different effects on different lakes. For lakes with greater glacier coverage in the watershed, higher temperatures promote more ice meltwater in the watershed, and bring more surface runoff water recharge to the lakes; for lakes with smaller glacier coverage in the basin, the main effect of increased temperature on the lakes is to increase the water output of the lakes due to evapotranspiration. 

## Data Availability

The CryoSat-2 Level 2 Baseline-D product data presented in this study are openly available from the European Space Agency at https://earth.esa.int/eogateway/missions/cryosat/data (accessed on 29 October 2021). The Hydroweb data presented in this study are openly available in Hydroweb website at https://hydroweb.theia-land.fr/ (accessed on 1 November 2021). The fifth generation of ECMWF Atmospheric Reanalysis of Global Climate (ERA5) data presented in this study are openly available in ECMWF at https://cds.climate.copernicus.eu/ (accessed on 9 March 2022).
